# Cross-Sector Antimicrobial Resistance and Virulence in *Enterococcus* spp. from Humans, Animals and the Environment

**DOI:** 10.3390/antibiotics15070657

**Published:** 2026-07-02

**Authors:** Nicolau Fialho, Joana Monteiro Marques, Maria Teresa Barreto-Crespo, Teresa Semedo-Lemsaddek

**Affiliations:** 1 Centre for Interdisciplinary Research in Animal Health (CIISA), Faculty of Veterinary Medicine, University of Lisbon, 1300-477 Lisbon, Portugal; 2 Associate Laboratory for Animal and Veterinary Sciences (AL4AnimalS), 1300-477 Lisbon, Portugal; 3 Faculty of Sciences, University of Lisbon, 1749-016 Lisbon, Portugal; 4 National Institute of Agricultural and Veterinary Research, IP (INIAV), 2780-157 Oeiras, Portugal; 5 cE3c—Centre for Ecology, Evolution and Environmental Changes & CHANGE, Global Change and Sustainability Institute, Faculty of Sciences, University of Lisbon, 1749-016 Lisbon, Portugal; 6 iBET—Instituto de Biologia Experimental e Tecnológica, 2780-157 Oeiras, Portugal; 7 Instituto de Tecnologia Química e Biológica António Xavier (ITQB), 2780-157 Oeiras, Portugal; 8 Biosystems & Integrative Sciences Institute (BioISI), Faculty of Sciences, University of Lisbon, 1749-016 Lisbon, Portugal

**Keywords:** *Enterococcus*, antimicrobial resistance, vancomycin-resistant *Enterococcus* (VRE), One Health, human–animal–environment interface

## Abstract

Background/Objectives: Antimicrobial resistance is a major public health concern requiring integrated surveillance across human, animal, and environmental sectors. *Enterococcus* spp. are widely distributed opportunistic bacteria with the capacity to acquire and disseminate resistance and virulence determinants. This study aimed to characterize species distribution, phenotypic antimicrobial resistance, resistance genes, and virulence-associated traits in *Enterococcus* spp. from One Health sources. Methods: Enterococci were recovered from 66 samples collected in Lisbon, Portugal, between late 2022 and early 2024, including healthy and sick humans, healthy and sick animals, canteen food, surface water, and public transport surfaces. RAPD-PCR was used to assess genetic diversity among 90 isolates and select 74 representative enterococci. Species identification, resistance gene screening, and virulence gene detection were performed by PCR. Antimicrobial susceptibility was assessed by disk diffusion against 12 antibiotics using CLSI criteria. Vancomycin resistance was further evaluated by agar dilution and Etest when applicable. Results: *Enterococcus faecalis* predominated, representing 63/74 isolates (85.1%), followed by *Enterococcus faecium* (5/74, 6.8%) and other *Enterococcus* spp. (6/74, 8.1%). Antibiotic resistance was detected across all One Health sectors. Sick Human isolates showed higher resistance than Healthy Human isolates. *E. faecium* showed higher resistance than *E. faecalis*, and all *E. faecium* isolates across sectors were multidrug-resistant. Rifampicin resistance was frequent, as was quinupristin-dalfopristin resistance (excluding intrinsically resistant *E. faecalis*), while linezolid resistance was not detected. Resistance and virulence determinants were distributed across sectors. Conclusions: Enterococci from human, animal, and environmental sources carried antimicrobial resistance and virulence-associated traits. These findings support integrated One Health surveillance to monitor resistant enterococci across interconnected reservoirs.

## 1. Introduction

Antimicrobial resistance (AMR) is a major threat to public health, reducing the effectiveness of antimicrobial therapy and increasing morbidity and mortality worldwide [1]. The global burden of AMR is substantial, with an estimated 4.95 million deaths associated with bacterial AMR, and 1.27 million directly attributable deaths [2]. This growing burden threatens the continued effectiveness of antimicrobial therapy and increases the difficulty of treating common infections [3].

Addressing the shared threat posed by AMR requires an integrated approach across human, animal and environmental sectors, as formalized by the One Health framework [3]. The concept recognizes that selection pressures in healthcare, agriculture and the wider environment interact to shape resistance patterns [4,5]. Effective surveillance and control therefore require coordinated action linking public health, veterinary medicine and environmental science and policies.

Enterococci are ubiquitous bacteria with a broad ecological distribution. They colonize the gastrointestinal tracts of wild and domestic animals and occur in soils, plants, insects, food products, hospital environments and the human microbiota [6,7,8,9,10,11,12,13,14]. In clinical settings, enterococci have emerged as opportunistic pathogens and are a major cause of healthcare-associated infections, including urinary tract infections, bacteremia, and infective endocarditis [15].

*E. faecalis* and *E. faecium* account for approximately 90 to 95% of enterococcal infections, while other species, including *Enterococcus hirae*, *Enterococcus gallinarum*, and *Enterococcus casseliflavus*, occur less frequently [16].

Enterococci exhibit intrinsic resistance to third-generation cephalosporins and aminoglycosides [17] and can also acquire virulence factors and Antimicrobial Resistance Genes (ARGs) through horizontal gene transfer events, including determinants conferring resistance to last resort antimicrobials [16].

Vancomycin-resistant *Enterococcus* (VRE) represent a major clinical concern, particularly vancomycin-resistant *E. faecium* (VREfm), which is frequently associated with hospital outbreaks [17,18]. This organism is classified by the World Health Organization as a high-priority pathogen, and is included in the ESKAPE group of pathogens composed of *E. faecium*, *Staphylococcus aureus*, *Klebsiella pneumoniae*, *Acinetobacter baumannii*, *Pseudomonas aeruginosa*, and *Enterobacter* spp. [17,19].

Vancomycin resistance in enterococci is mediated by the *van* gene cluster. The *vanA* and *vanB* genotypes are most frequently detected in *E. faecium* [17,20]. Vancomycin-variable *Enterococcus* (VVE) isolates appear phenotypically susceptible to vancomycin while harboring *vanA*, but express resistance after exposure to glycopeptides [17,21].

As VRE prevalence has increased, linezolid has been used as a last resort oxazolidinone due to its activity against Gram-positive pathogens through inhibition of protein synthesis [22,23]. However, resistance to linezolid emerged within one year of its introduction and despite remaining relatively low, has been increasing among clinical isolates [17].

Resistance patterns in enterococci are shaped by antimicrobial selection pressures operating across healthcare, agriculture, aquaculture, and environmental settings [24,25]. Comparative and genomic studies have demonstrated differences in species composition and resistance profiles across sources, complicating interpretation when surveillance remains restricted to individual sectors [17,26,27].

In addition to antibiotic resistance, the assessment of virulence determinants is essential for understanding the pathogenic potential and dissemination of enterococcal lineages across the One Health continuum [17]. Virulence factors such as *agg*, *cylA*, *esp* and *gelE* are commonly identified in enterococci among humans [28], animals [13,16,29,30,31,32], and the environment [33,34,35] across the world.

Enterococci have traditionally been studied within separate human, animal, or environmental sectors, with limited interdisciplinary integration across these domains [36,37]. This compartmentalized approach constrains understanding of resistance reservoirs and transmission pathways across the One Health continuum [17,26,27].

Despite recognition of these limitations, integrated studies comparing species distribution and resistance profiles across sectors remain limited. Existing surveillance and review studies frequently rely on sector-specific datasets, which restrict inference on the dissemination of resistance determinants between interconnected sources [17,26,27].

This study aimed to characterize the species distribution, as well as the phenotypic and genotypic AMR and virulence profiles, of *Enterococcus* spp. isolated from human, animal, and environmental sources within a One Health framework, in the Lisbon metropolitan area, Portugal. The Lisbon metropolitan area was selected due to its geographical proximity, high degree of urban interconnectedness, and the growing epidemiological relevance of VREfm in Portugal, where increasing levels of VRE have been reported in recent years [38]. We hypothesized that isolates from these interconnected reservoirs would exhibit overlapping AMR and virulence determinants, supporting the ecological interconnectedness of enterococci across One Health sectors.

## 2. Results and Discussion

### 2.1. Microbial Collection

Enterococci were isolated from a total of 66 samples from One Health sources (Humans, Animals and Environment), including Healthy (n = 14) and Sick (n = 17) humans with varied enterococcal infections, Healthy (n = 10) and Sick outpatient (n = 12) animals (non-enterococcal infection), Canteen Food (n = 9), Surface Water (n = 1) and Public Transport Surfaces (n = 3). Clinical isolates from enterococcal infections included genitourinary infections (urine, n = 7), tissue infections (biopsy, n = 3), purulent infections (pus, n = 3), systemic infections (blood, n = 2), and unknown infection sites (n = 2). Following isolation in Slanetz-Bartley agar (SBA) and SBA supplemented with vancomycin (SBAvan), culture purity and presumptive genus-level identification were confirmed by successive subcultures on SBA and Bile-Esculin Azide agar (BEA). From this initial purification, 90 isolates were retrieved, 35 from the Human sector, 27 from the Animal sector, and 28 from the Environment sector.

Given the ubiquity of enterococci across One Health sectors, the first analytical objective of this study was to assess microbial diversity and select representative isolates for further characterization. This approach aimed to reduce redundancy while preserving the diversity of enterococcal populations across sectors (humans, animals, and environment) and respective subsectors, ensuring balanced representation in subsequent analyses.

Random Amplification of Polymorphic DNA-Polymerase Chain Reaction (RAPD-PCR) profiles generated with primers OPC15 and (GTG)_5_ were combined to construct a composite dendrogram based on the average similarity of both analyses. The mean reproducibility of the method was 76.6%, which was adopted as the similarity threshold for cluster definition and is indicated in Supplementary Figure S1.

Following RAPD-PCR clustering and application of the predefined selection criteria, a final subset of representative enterococcal isolates was established for downstream analyses. This collection comprised a total of 74 isolates, including 31 from the Human sector, 22 from the Animal sector and 21 from the Environment sector. Considering subsector distribution, it includes Healthy Humans (n = 14) and Sick Humans (n = 17), Healthy Animals (n = 10) and Sick Animals (n = 12), Canteen Food (n = 9), Surface Water (n = 6), and Public Transport Surfaces (n = 6). Of these isolates, 5 from Healthy Humans, 1 from Sick Animals and 2 from Canteen Food were retrieved from SBAvan plates.

### 2.2. Molecular Species Identification

**Figure 1:**
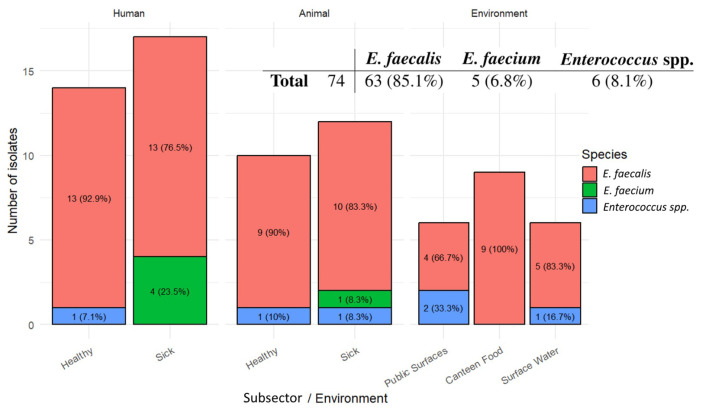
Distribution of *E. faecalis*, *E. faecium* and other *Enterococcus* spp. across One Health sectors and subsectors.

Species identification was performed by Polymerase Chain Reaction (PCR). Species distribution results, by sector and subsectors, are summarized in Figure 1 and detailed by isolate in Supplementary Table S1. Isolates that could not be assigned to *E. faecalis*, *E. faecium*, or *Enterococcus durans* were tested for the genus and classified as *Enterococcus* spp.

Among the 74 representative isolates analyzed, *E. faecalis* was the dominant species, accounting for 63 isolates (85.1%), followed by *E. faecium* with 5 isolates (6.8%), while 6 isolates (8.1%) remained non-determined at the species level and were identified at the genus level. *E. durans* was not identified in our collection. *E. faecalis* predominated across all One Health sectors, although its relative dominance was lower in Environmental samples, where other *Enterococcus* spp. were more frequently recovered, particularly from Surface Water and Public Transport Surfaces. This broader diversity is consistent with the heterogeneous nature of environmental matrices and their exposure to multiple contamination sources [39,40,41,42].

Subsector-specific patterns were evident. In the Human sector, *E. faecalis* predominated in both Healthy and Sick isolates, while *E. faecium* was detected only among Sick Human isolates, supporting its recognized association with hospital-associated infections and clinical settings [43,44,45]. A similar pattern was observed in Animals, where *E. faecalis* remained dominant and *E. faecium* was detected in only one Sick Animal isolate. This aligns with studies reporting *E. faecalis* as frequent in animal hosts, while variation in other species, including *E. hirae* and *Enterococcus cecorum*, reflects host species, age, and agricultural practices [26,46,47,48].

The predominance of *E. faecalis* is consistent with its ecological versatility as a generalist commensal, its frequent occurrence in the gastrointestinal tract of humans and animals, and its capacity to persist in soil, water, sediments, plants, food products, and other environmental compartments [11,49,50,51,52,53]. Enterococcal tolerance to environmental stressors, oxidative stress adaptation, virulence traits, and biofilm formation further support persistence across host-associated and abiotic environments [9,15,17,20,36]. Nevertheless, the distribution of enterococci remains context dependent, as hospital settings have seen an increasing role of multidrug-resistant *E. faecium*, particularly in bloodstream infections and in association with resistance to ampicillin and vancomycin [17,18,50,51,54,55]. Culture-based isolation may also have favored the recovery of *E. faecalis*, as commonly used media select for bile tolerance, esculin hydrolysis, and robust growth, potentially underrepresenting less tolerant species [31,56,57,58].

### 2.3. Antibiotic Resistance

#### 2.3.1. Phenotypic Resistance Assay

Antimicrobial susceptibility testing (AST) was performed using the disk diffusion method. Results were interpreted according to breakpoints established by the Clinical and Laboratory Standards Institute (CLSI). Although the European Committee on Antimicrobial Susceptibility Testing (EUCAST) criteria are geographically appropriate for a European dataset, EUCAST breakpoints were unavailable for several antibiotics included in this panel (specifically quinupristin-dalfopristin, rifampicin, and chloramphenicol) and, where available, differ from CLSI in both disk concentrations and interpretive thresholds, limiting direct comparability across compounds and studies. Given these constraints and the broader availability of published reference data using CLSI, this system was applied throughout. The use of CLSI rather than EUCAST is acknowledged as a limitation and should be considered when comparing results with European surveillance datasets.

**Figure 2:**
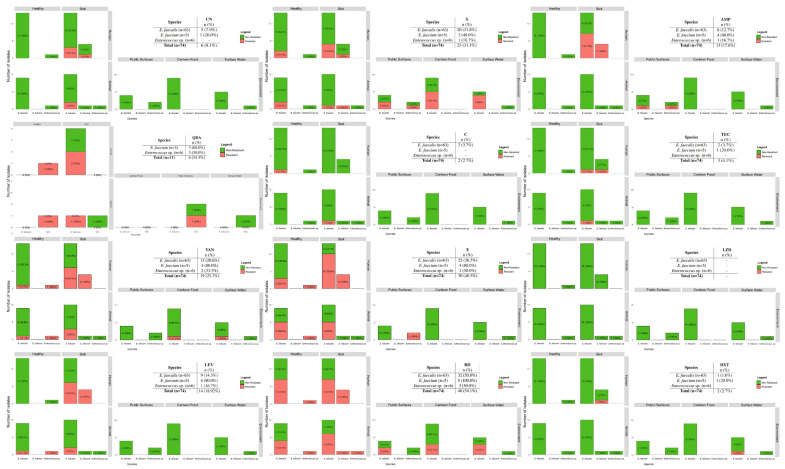
Distribution of *Enterococcus* spp. exhibiting resistance to each tested antibiotic by species and subsector.

Phenotypic resistance was assessed for each antibiotic by One Health sector and subsector. The distribution of resistance by enterococcal species and One Health subsector is plotted in Figure 2, while resistance frequencies by sector and subsector are presented in Table 1. Resistance frequencies were calculated considering only isolates classified as resistant according to CLSI breakpoints. Antibiotic resistance profiles for individual isolates are provided in Supplementary Table S1.

Across the One Health framework, higher resistance prevalences were consistently observed among isolates from Sick Humans, followed by Sick Animals, whereas isolates from Healthy Humans, Healthy Animals, and Environmental subsectors generally exhibited lower resistance levels. Exceptions were noted for streptomycin, quinupristin-dalfopristin, and doxycycline, for which elevated resistance was detected in specific Environmental subsectors. Overall, *E. faecium* displayed higher resistance rates than *E. faecalis* across most antibiotics, reflecting its recognized role as a hospital-adapted and Multidrug-Resistant (MDR) lineage [18,50,51]. The main exception was chloramphenicol, for which resistance was detected exclusively in *E. faecalis*. These patterns broadly align with existing literature, supporting a gradient of resistance intensity from clinical to non-clinical settings while underscoring species-specific differences in resistance burden.

Aminoglycoside resistance displayed marked heterogeneity across sectors and between compounds. Resistance to gentamicin was uncommon overall and was primarily detected in Sick patients, particularly among Sick Humans. This pattern aligns with the historical role of gentamicin in severe enterococcal infections, where synergy with cell-wall active agents was established and exploited in combination therapy [50,59]. Following recognition of toxicity at high doses and the introduction of alternative agents, gentamicin use declined, although renewed interest has emerged in the context of MDR infections and combination regimens [60,61,62,63]. Consistent with this clinical selectivity, little to no gentamicin resistance was detected in several environmental and food-associated contexts referenced in the literature, including transport-associated surfaces, ready-to-eat foods, cheeses, and surface water [34,64,65,66,67,68], although earlier Portuguese drinking-water data reported high-level gentamicin resistance in a subset of isolates [69]. In contrast to the clinically restricted distribution of gentamicin resistance, resistance to streptomycin was widespread across the One Health continuum in the present dataset and Public Transport Surfaces; and represented one of the most prevalent resistance phenotypes observed. Similar resistance has been repeatedly reported across healthy and clinical human isolates, diverse animal hosts, foods, and environmental waters [28,34,44,47,68,69,70,71,72,73,74,75]. This broad distribution is consistent with the long-standing use of streptomycin in human medicine and its documented use in animal settings, including historical growth promotion, which together have supported the persistence and dissemination of high-level streptomycin resistance determinants across multiple compartments [26,42,50,66,76]. Taken together, aminoglycoside resistance patterns illustrate contrasting selection histories: gentamicin resistance appears more tightly linked to clinical exposure, while streptomycin resistance reflects widespread dissemination across multiple One Health reservoirs [50,77].

Resistance to ampicillin showed a clear sectoral and species-associated pattern. No resistance was detected in Healthy Humans, consistent with reports from Portugal and Tunisia, where resistance remains low in *E. faecalis* and limited in *E. faecium* [28,74]. In contrast, resistance increased markedly in Sick Humans, particularly in *E. faecium*, in line with its established association with clinical infections [44,78]. No resistance was observed in the Animal sector, which contrasts with studies reporting moderate to high prevalence in livestock and companion animals [47,68,79]. This discrepancy may partly reflect the limited sample size in the animal subsector of the present study, which may have been insufficient to detect resistance at the lower prevalence levels described in some populations [75,80]; this should be considered a constraint when interpreting the absence of resistance in this sector. Environmental sources exhibited low prevalence overall, with sporadic detection on Public Transport Surfaces and absence in Food and surface Water, consistent with reports from food and environmental matrices [34,64,66,67,68,69,70]. These patterns reflect the role of ampicillin as a first-line agent and the preferential accumulation of resistance in hospital-adapted *E. faecium*, while *E. faecalis* remains largely susceptible [50].

Resistance to glycopeptides showed a marked concentration in clinical settings. Teicoplanin resistance was restricted to Sick Humans and Animals, with no detection in Healthy or Environmental subsectors, consistent with reports describing low prevalence outside clinical contexts [73,74,75]. In contrast, vancomycin resistance reported by disk-diffusion was more widely distributed but remained primarily associated with Sick Humans and Animals, with most resistant isolates belonging to *E. faecium*, frequently classified as VREfm, in line with its recognized dominance in clinical infections [26,81,82]. Lower prevalence in Healthy Humans and Animals agrees with community and livestock studies reporting limited vancomycin resistance [43,73,74,75,83]. Environmental sources showed low prevalence, with absence on Public Transport Surfaces and sporadic detection in Canteen Food and Surface Water, although higher levels have been reported in wastewater-impacted environments, particularly involving *E. faecium* [19,34,67]. These patterns reflect the extensive clinical use of glycopeptides and the selective pressure driving the emergence and persistence of vancomycin-resistant enterococci. The historical use of avoparcin, a glycopeptide antibiotic used as a growth promoter in European livestock prior to its ban in 1997, is well recognized as a key driver in the early selection and environmental establishment of VRE reservoirs, contributing to the persistence of resistance determinants in animal and environmental compartments observed to this day [76,84]. Vancomycin resistance was confirmed by Etest in 19 isolates considered resistant in both AST and agar dilution methods, with Minimum Inhibitory Concentrations (MIC) ranging from 2 to 256 μg/mL. Vancomycin Etest MICs among resistant isolates were distributed as follows: 2 μg/mL (n = 4, 1 from Sick Humans, 1 from Sick Animals, 1 from Healthy Animals, 1 from Surface Water), 4 μg/mL (n = 5, 1 from Healthy Animals, 2 from Sick Animals, 1 from Canteen Food), 8 μg/mL (n = 1 from Healthy Humans), 64 μg/mL (n = 1 from Sick Humans), and ≥ 256 μg/mL (n = 8 from Sick Humans) (detailed in Supplementary Table S1. The ten isolates with MIC values below the CLSI resistance threshold for vancomycin (32 μg/mL) were reclassified and interpreted as susceptible or intermediate VVE isolates and were not considered in the VAN-MIC column on Table 1. High-level vancomycin resistance (MIC ≥ 256 μg/mL) was restricted to clinical isolates, being detected in 8 of the 19 enterococci previously classified as VRE and recovered from three different healthcare entities (two hospitals and a clinical laboratory). This finding is consistent with the recognized predominance of VRE in healthcare-associated settings and hospitalized patient populations. An overall prevalence of 12.1% (9/74) was observed, with all resistant isolates originating from the Human sector, primarily from clinical infection samples. At the European level, EARS-Net surveillance highlights the continued clinical importance of VREfm, with a population-weighted mean resistance prevalence of 16.5% among invasive isolates in the EU/EEA in 2024 and an estimated incidence of 1.96 bloodstream infections per 100,000 population [38]. Similarly, Portugal has reported increasing levels of VREfm, rising from 4.3% to 11.0% from 2018 to 2022 among invasive isolates [85]. However, in our study, only 37.5% of high-MIC VRE were *E. faecium*, whereas the remainder were *E. faecalis*. This finding contrasts with EARS-Net surveillance trends, where vancomycin resistance is predominantly associated with *E. faecium* clinical isolates, suggesting potential differences in the epidemiology of VRE within the study population and sampling methods.

Resistance to erythromycin was widespread across sectors, with higher prevalence in Sick Human and Animal isolates and lower but persistent levels in Healthy and Environmental subsectors. In Humans, resistance increased markedly in Sick isolates, consistent with reports of elevated macrolide resistance in clinical enterococci, while remaining lower in Healthy individuals [28,44,74]. In the Animal sector, high resistance was observed in both Healthy and Sick isolates, aligning with reported variability linked to host species and antimicrobial exposure [46,73,75]. Environmental sources showed lower prevalence, although resistance was detected on Public Transport Surfaces, supporting dissemination beyond clinical and agricultural settings, as described in urban and aquatic environments [66,67,68,70]. Joint Wald tests were performed to assess the relationship between each sector for each antibiotic. For erythromycin, the Environmental sector had significantly lower resistance counts compared to both the Human (*p* = 0.034) and the Animal (*p* = 0.039) sectors. Species-specific patterns were evident, with *E. faecalis* accounting for most resistant isolates, consistent with the widespread distribution of macrolide resistance determinants such as the *erm(B)* gene, frequently associated with mobile genetic elements and co-localized with other ARGs [46,50]. These patterns reflect sustained selective pressure driven by historical and ongoing macrolide use across sectors, supporting the persistence and dissemination of resistance within a One Health framework.

Linezolid resistance was not detected across any sector or subsector, with all Human, Animal, and Environmental isolates remaining phenotypically susceptible, including those from sick hosts. This finding aligns with most reports describing low prevalence of resistance in enterococci, typically below 5% for both *E. faecalis* and *E. faecium* [16,17,50]. However, higher prevalence has been reported in specific contexts, including fecal carriage in Switzerland, hospital isolates in Romania, companion animals, and wastewater-associated environments [19,44,47,86], indicating that resistance emergence is context-dependent and influenced by local selective pressures. The sustained efficacy of linezolid reflects its restricted use as a last-resort agent for multidrug-resistant Gram-positive infections, including VRE [50]. Resistance, when present, is mediated by target site mutations and transferable determinants associated with cross-resistance to other antimicrobial classes [17,46,87]. Although not approved for veterinary or agricultural use, the detection of such determinants in animal and environmental isolates suggests indirect selection via co-located ARGs. These results support the continued effectiveness of linezolid, while highlighting the need for ongoing phenotypic and genotypic surveillance across One Health sectors.

Resistance to chloramphenicol was uncommon overall, with very low prevalence and resistant isolates restricted to Healthy subsectors. No resistance was detected among Sick Human or Sick Animal isolates, or in any Environmental subsector. Detection of resistance in Healthy Animal isolates contrasts with some reports of complete susceptibility but remains within the variability described across host species [73,75,88]. This pattern is consistent with contemporary studies reporting low chloramphenicol resistance in enterococci from distinct sources, particularly in Europe [28,74,89], and likely reflects reduced clinical use following recognition of severe toxicity, including aplastic anemia, and restrictions in food-producing animals [90,91,92,93]. Notably, chloramphenicol resistance genes are often transferable and co-located with determinants conferring resistance to other antimicrobial classes, allowing their maintenance through co-selection even in the absence of direct drug exposure [46,94].

Resistance to levofloxacin showed a clear sectoral pattern, with high prevalence among Sick Human isolates, lower but detectable resistance in Animal isolates, and no resistance in Healthy Human or Environmental subsectors. In Sick Humans, resistance affected all *E. faecium* and a substantial proportion of *E. faecalis*, consistent with reports of elevated fluoroquinolone resistance in enterococci recovered from infections, particularly *E. faecium* [26,78]. In Animals, resistance remained within the broad range reported for livestock and companion animals, where prevalence varies with host species and antimicrobial exposure [47,72,75]. The absence of resistance in Canteen Food, Surface Water, and Public Transport Surfaces aligns with food surveillance studies reporting minimal resistance [66,70,95], although higher levels have been described in aquatic environments influenced by agricultural or urban run-off, especially involving *E. faecium* [67,96]. These patterns reflect the extensive use of fluoroquinolones in human medicine and historical or extra-label use in animals, with regulatory restrictions potentially having reduced selection pressure in non-human sectors [75,97,98,99,100]. Continued monitoring remains important given the clinical relevance of fluoroquinolones and their classification as critically important antimicrobials for human health [101].

Resistance to rifampicin was among the highest observed in this study, with more than half of all isolates showing non-susceptibility and resistance detected across all sectors. Prevalence was particularly high among Sick Human and Animal isolates, and most pronounced in *E. faecium*, where all isolates were resistant. Human-sector resistance exceeded several European and international reports, where clinical enterococcal resistance typically ranges from approximately 20% to 40% [44,102]. The higher resistance rates observed here may reflect the composition of the clinical sample, which was enriched for severely ill or bacteremic patients at a tertiary referral center, populations known to harbor higher proportions of MDR *E. faecium* [102]. Regional prescribing patterns, including the use of rifampicin as part of combination regimens for difficult-to-treat infections such as VREfm endocarditis, may also have contributed to elevated local selection pressure [103]. The higher resistance in *E. faecium* compared with *E. faecalis* is consistent with published species-specific patterns [102]. In Animals, resistance fell within the broad range reported for livestock and companion animals [47,48,89]. Environmental resistance, particularly in Surface Water, closely matched findings from municipal wastewater effluents and receiving rivers, supporting the view that rifampicin resistance in environmental enterococci primarily reflects contamination from human sources rather than direct environmental selection [50,104]. The high cross-sector prevalence observed supports continued surveillance of rifampicin resistance across One Health settings.

Resistance to quinupristin-dalfopristin was among the highest observed, with its prevalence being only documented for *E. faecium* and other *Enterococcus* spp., as *E. faecalis* exhibits intrinsic resistance to streptogramins and is not a clinical target for this antibiotic. The therapeutically relevant resistance burden therefore lies among *E. faecium* and isolates from other *Enterococcus* spp., for which acquired resistance is of genuine clinical concern [50]. With this distinction in mind, resistance in Human isolates aligns with reports from hospital-associated enterococci in Portugal and elsewhere [74], while Animal-sector resistance falls within the range described for livestock and companion animals [47,75]. Although quinupristin-dalfopristin is not approved for veterinary use, historical use of the related streptogramin virginiamycin as a growth promoter likely contributed to the selection of resistant enterococci in animal-associated reservoirs [50,105]. Environmental resistance, detected on Public Transport Surfaces, likely reflects dissemination from human and animal sources, with resistance not being found in other Environmental subsectors, most likely due to the reduced sample size [65,70,71,106]. Given its role as a therapeutic option for VREfm and MDR Gram-positive infections, the widespread acquired resistance in *E. faecium* warrants continued One Health surveillance of streptogramin resistance [50,52,107].

Resistance to doxycycline was infrequent in this dataset, with resistant isolates restricted to Sick Humans and Surface Water, and no resistance detected in Healthy Humans, Animals, Public Transport Surfaces, or Canteen Food. This diverges substantially from the broader literature, where tetracycline resistance is commonly reported at moderate to high prevalence across One Health sectors [28,48,74]. In Sick Humans, resistance affected only a minority of *E. faecalis*, whereas studies using tetracycline as a proxy report higher resistance in both carriage and clinical isolates [26,28,44]. The complete absence of resistance in Animals also contrasts with reports of widespread tetracycline resistance in livestock and companion animals [47,80,88]. These discrepancies likely reflect a combination of factors: the relatively limited sample sizes in several subsectors of this study, the host composition of the animal cohort, and importantly, methodological differences between doxycycline- and tetracycline-specific testing, including the marked divergence in interpretive breakpoints between CLSI and EUCAST for the tetracycline class, where CLSI breakpoints are routinely higher, typically by around 4-fold, producing substantially different susceptibility classifications for the same isolate depending on the system applied [108], and the documented variability in genotype–phenotype concordance for doxycycline specifically in *Enterococcus*, where false-positive resistance rates of up to 27% have been reported due to variability in the *tet(M)* sequence [109], compounded by known inter-method discrepancies between disk diffusion and agar dilution for doxycycline when testing *E. faecalis* and *E. faecium* [110]. Furthermore, tetracycline is more frequently used in clinical and veterinary settings and has historically shown higher resistance rates; doxycycline-specific testing may therefore underestimate the true burden of tetracycline-class resistance. Environmental resistance detected only in Surface Water is consistent with reports linking tetracycline resistance to aquatic environments influenced by agricultural run-off and wastewater discharge [67,69]. These anomalous findings should be interpreted cautiously and underscore the importance of standardized compound-specific testing in One Health surveillance programs.

**Figure 3:**
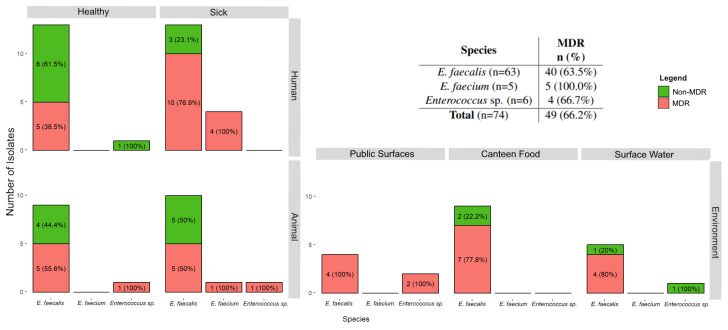
Distribution of *Enterococcus* exhibiting multidrug resistance. Percentages were calculated based on the total number of MDR isolates within each One Health subsector. The Total row represents the overall percentage of MDR across all enterococci.

The AMR profiles of the *Enterococcus* isolates were categorized to identify MDR phenotypes. MDR was defined as non-intrinsic, non-susceptibility to at least one agent in three or more antimicrobial classes [60]. Therefore, both intermediate/susceptible with increased exposure (I) and resistant (R) categories were grouped as non-susceptible for MDR classification and analysis. In addition, the analysis did not include QDA resistance in *E. faecalis*. The prevalence of MDR is presented by enterococcal species and subsector in Figure 3. Individual isolates classified as MDR are identified in Supplementary Table S1.

Multidrug-resistant isolates were detected across all One Health sectors, with the highest proportions observed among Sick isolates and Public Transport Surfaces. All *E. faecium* isolates were classified as MDR; while this finding is consistent with the recognized clinical association between this species and multidrug resistance [44,74], it should be noted that the *E. faecium* sample size in this study was limited, and the finding should be interpreted accordingly rather than as a precise population estimate. In Humans, MDR prevalence was higher among isolates from Sick Humans compared to Healthy humans, consistent with studies reporting greater multidrug resistance in enterococci recovered from infections, particularly *E. faecium* [28,44,74]. It should be noted that the Sick Humans subsector comprised isolates recovered from a variety of clinical infection types and individuals with different underlying comorbidities, which may have acted as confounding factors in the observed resistance patterns.

In Animals, MDR was common in both Healthy and Sick enterococci and fell within the range reported for livestock and companion animals, where prevalence varies with antimicrobial exposure, host species, production system, and geography [47,75,80]. Environmental MDR was also frequent, with all Public Transport Surfaces isolates classified as MDR and high prevalence in Canteen Food and Surface Water, consistent with reports identifying community surfaces, river water, and wastewater-impacted environments as reservoirs of clinically relevant resistance phenotypes [34,58,69]. The highest numbers of non-susceptibilities were concentrated in Sick Human isolates, while only two isolates, both *E. faecalis* from Healthy Animals, remained fully susceptible, supporting the role of enterococci as reservoirs and disseminators of AMR within the One Health framework.

#### 2.3.2. Genotypic Resistance Determinants

**Figure 4:**
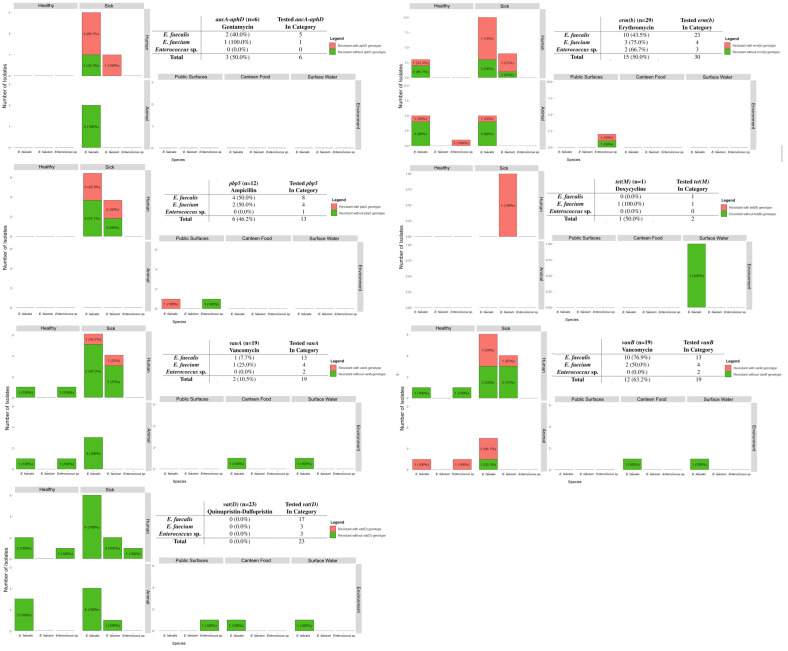
Distribution of *Enterococcus* carriage of genetic resistance determinants (*aacA-aphD*, *erm(B)*, *pbp5*, *tet(M)*, *vanA*, *vanB*, *vanC*, *vat(D)*), by enterococcal species in distinct subsectors.

A panel of ARGs (*aacA-aphD*, *erm(B)*, *pbp5*, *tet(M)*, *vanA*, *vanB*, *vanC*, *vat(D)*) was tested by Multiplex PCR using the primers and conditions described below in Section 3.4.2. Isolates were selected for testing according to their phenotypical resistance profile, with the objective of providing a targeted phenotypic-genotypic overview of the most clinically and epidemiologically relevant determinants. The results obtained are summarized in Figure 4, and Supplementary Table S1 lists the ARGs identified for each isolate.

The *aacA-aphD* gene, a major determinant of High-Level Gentamicin Resistance (HLGR) and resistance to other aminoglycosides [41], was detected exclusively in gentamicin-resistant isolates from the Sick Human subsector. Overall, *aacA-aphD* was identified in 75% of tested Sick Human isolates, including 66.7% of *E. faecalis* and all tested *E. faecium*. This prevalence closely aligns with a report from Sick Human isolates, where approximately 64% of HLGR isolates carried *aacA-aphD* [111]. In contrast, the gene was not detected in isolates from Sick Animals, consistent with the literature showing species-dependent variability and generally low carriage outside clinical human settings [47].

Resistance to macrolides and related compounds was assessed through detection of the *erm(B)* gene, which mediates resistance to macrolide, lincosamide, and streptogramin B antibiotics and is frequently associated with mobile genetic elements [105,112]. The *erm(B)* gene was detected across all One Health sectors, with approximately half of the tested isolates carrying this determinant. In the Human sector, *erm(B)* carriage was higher among Sick Human isolates, where 70% of *E. faecalis* and 75% of *E. faecium* were positive, corresponding to an overall prevalence of 71%. These values are consistent with reports from Portuguese studies of erythromycin-resistant enterococci [111]. Lower prevalences were observed among Healthy Humans and Animal subsectors, although detection across these groups aligns with previous reports from Southern Europe [73,74]. In Environmental subsectors, *erm(B)* was detected sporadically in Public Transport Surfaces isolates, in agreement with prior findings from public transportation environments in Portugal [68].

Resistance to β-lactams was assessed through the detection of mutations in the chromosomally encoded *pbp5* gene, which confers reduced susceptibility to penicillins such as ampicillin and to cephalosporins, particularly in *E. faecium* [113]. The *pbp5* gene was broadly distributed across subsectors and species, being present in 42.9% of tested *E. faecalis* isolates and in 50% of tested *E. faecium* isolates from Sick Humans. The gene was also detected in half of the tested Public Transport Surfaces isolates. This widespread distribution is consistent with previous reports across human, animal, and environmental sources [36,47,68,74,96,114]. While there are reports of universal carriage of *pbp5* mutations among ampicillin-resistant *E. faecium* from clinical settings [36], the lower prevalence observed here likely reflects limited sampling. However, while the presence of the *pbp5* gene was assessed in this study, it is important to emphasize that β-lactam resistance arises from specific mutations and allelic variations in *pbp5*, rather than its presence. Consequently, the results presented here reflect gene prevalence rather than resistance potential.

The *tet(M)* gene, the most prevalent tetracycline resistance determinant in *Enterococcus* spp. and commonly associated with mobile elements of the Tn916/Tn1545 family [59,105], was detected in only one of the two tested isolates. This isolate belonged to *E. faecium* from the Sick Human subsector, while the second tested isolate, recovered from Surface Water and identified as *E. faecalis*, did not carry *tet(M)*. This low resistance and *tet(M)* detection contrasts with numerous reports describing widespread carriage of *tet(M)* in Human, Animal, and Environmental sectors, including both Healthy and Sick hosts and diverse environmental matrices [28,47,68,69,70,74,75,115,116].

Acquired glycopeptide resistance was assessed in isolates that exhibited phenotypic resistance to vancomycin by AST, through the detection of the *vanA* and *vanB* gene clusters, while intrinsic low-level resistance was evaluated via *vanC* operon screening. The *vanA* gene, which confers resistance to vancomycin and teicoplanin and is typically associated with mobile transposons [59,105], was detected exclusively in the Sick Human subsector. Within this group, *vanA* was identified in 25% of tested *E. faecium* and 16.7% of tested *E. faecalis* isolates. This distribution is consistent with the dominance of *vanA* among VRE in European hospitals [117]. No *vanA*-carrying isolates were detected among Healthy Humans, Animals, or Environmental subsectors, in line with reports of low or absent carriage in community, animal, and most environmental settings [28,74,80,118,119]. Environmental detection of *vanA* has been mainly reported in hospital-impacted matrices, such as wastewater and downstream surface waters [34,120], which were not represented among the VRE isolates analyzed here.

The *vanB* gene cluster, an acquired determinant typically carried on conjugative transposons and conferring resistance to vancomycin but not teicoplanin [46,105], showed a broader distribution. Overall, 42.1% of vancomycin-resistant isolates carried *vanB*, spanning Human and Animal sectors. In Sick Humans, *vanB* was detected in approximately half of *E. faecalis* isolates and in a quarter of *E. faecium*. Similar moderate prevalences have been reported in European studies for Healthy Humans, yet contrasting with higher rates in some hospital-associated VREfm lineages and lower rates in community and animal reservoirs [121,122]. In the Animal sector, the high proportion of *vanB*-positive isolates observed contrasts with generally lower prevalences reported in the literature [80,123] and is likely influenced by the limited number of isolates tested. In the Environmental sector, the low prevalence observed is confirmed by the literature [34,70].

In contrast to the acquired operons, the *vanC* gene cluster, which is chromosomally encoded and confers intrinsic low-level resistance to vancomycin, was not detected in any isolates from this study. This finding is consistent with the established association of *vanC* with species such as *E. gallinarum*, and *E. casseliflavus/Enterococcus flavescens* [46,124], which were not specifically targeted in this study due to their relatively low prevalence and the characteristic yellow pigmentation typically exhibited by these species on agar media. No *vanC*-positive isolates were detected among *E. faecalis* or *E. faecium* in this study, in contrast to sporadic reports of interspecies transfer in wastewater-impacted environments [34].

The *vat(D)* gene, encoding a streptogramin A acetyltransferase and typically carried on mobile genetic elements [24,46,105], was not detected in any isolate analyzed. This absence is consistent with recent genomic and phenotypic surveys reporting very low prevalence of *vat(D)* among *Enterococcus* spp. from human, animal, and environmental sources [26,28,46,125,126,127]. The lack of detection in this study supports the view that *vat(D)* remains a rare determinant across the One Health continuum.

Overall, the distribution of ARGs observed in this study reflects a clear sectoral stratification for certain determinants, particularly those associated with high-level resistance and clinical selective pressure, such as *aacA-aphD* and *vanA*, which were confined to Sick Human isolates. In contrast, genes such as *pbp5*, *vanB*, and *erm(B)* displayed broader distributions across One Health sectors, consistent with their known mobility and persistence in both clinical and non-clinical reservoirs. Intrinsic resistance determinants, exemplified by *vanC*, remained restricted to non-*E. faecalis*/*non-E. faecium* species. Together, these findings support the view that while clinical settings act as focal points for the selection of high-impact resistance determinants, a substantial proportion of the resistome circulates across human, animal, and environmental compartments, supporting the relevance of integrated One Health surveillance.

### 2.4. Virulence Factor Production

#### 2.4.1. Hemolytic Activity

Hemolytic activity was assessed on blood agar plates supplemented with horse blood under anaerobic incubation. Horse blood was used instead of sheep blood based on previous findings from our group showing that horse erythrocytes are more susceptible to hemolysin-mediated lysis, and that β-hemolysis on horse blood under anaerobic conditions is significantly associated with the detection of *cyl* genes [128]. Anaerobic incubation was used to minimize oxidation-related erythrocyte lysis not directly attributable to enterococcal hemolysin activity [128].

Hemolytic activity results are listed for each isolate in Supplementary Table S1. Overall, β-hemolysis was detected in 20.3% of enterococcal isolates (15/74). Hemolytic activity was observed across all One Health sectors, with the highest proportion in Environmental isolates (23.8%, 5/21), followed by Human isolates (19.4%, 6/31) and Animal isolates (18.2%, 4/22). All β-hemolytic isolates were identified as *E. faecalis*, except for one Environmental isolate identified only at the genus level. Previous studies have reported variable prevalences of β-hemolysis among enterococci from One Health sources [129,130].

#### 2.4.2. Virulence Gene Screening

A virulence gene screening was carried out by multiplex PCR for *agg* (aggregation substance), *cylA* (cytolysin activator), *esp* (enterococcal surface protein) and *gelE* (gelatinase) genes. In general, *gelE* was the most frequently identified (39.2%, 29/74), followed by *esp* (36.5%, 27/74), *agg* (24.4%, 18/74) and *cylA* (17.6%, 13/74), although these prevalences changed when considering each sector.

The virulence gene screening results are listed for each isolate in Supplementary Table S1. Among the virulence genes screened per sector, *esp* was the most frequent in Human isolates, being observed in 58.1% (18/31), with most *esp*-positive isolates associated with infections (Sick Humans; 77.7%, 14/18). Another study on clinical and food isolates in Brazil also observed a higher prevalence of *esp* (68.4%) among human clinical isolates [131]. In our study, this gene was also present in 27.3% (6/22) of Animal enterococci and 14.3% (3/21) of Environment isolates. The moderate-to-high prevalence of *esp* in animals and lower prevalence in environmental-associated enterococci, such as food, is consistent with the literature [32,33,131]. Analysis of the *esp* gene using a joint Wald test revealed a significant association between the sector and gene prevalence, with a significant difference detected between the Environmental and Human sectors (*p* = 0.0492). No significant associations between sector and the prevalence of the remaining virulence genes were detected.

In contrast, *gelE* was the most prevalent gene in both Animal and Environment isolates, occurring in 40.9% (9/22) and 42.9% (9/21), respectively. Other authors reported a high prevalence of *gelE* in animal-associated enterococci [13,16,30,31,32] and environmental sources such as food (only in *E. faecalis*) [131]. Overall, *gelE* was also consistently detected across all sectors, including 35.5% (11/31) of Human isolates, which underscores its role as a fitness determinant rather than only an infection-associated gene.

As for the *agg* gene, it showed a similar prevalence to *gelE* among Human isolates (35.5%, 11/31), but was less frequent in Animal and Environment isolates, where it was detected in 18.2% (4/22) and 14.3% (3/21), respectively. This finding contrasts with previous reports stating a higher prevalence of *gelE* compared with *agg* among human clinical isolates [131], while *agg* was reported as the most prevalent (64.7%) virulence factor among enterococci isolated from wild animals [132].

The *cylA* gene was less prevalent overall, occurring in 22.6% (7/31) of Human isolates, 13.6% (3/22) of Animal isolates, and 14.3% (3/21) of Environment isolates. Among the seven *cylA*-positive human isolates, 71.4% were associated with infections, consistent with the known role of cytolysin in enhancing enterococcal virulence in infection models and the higher mortality observed in infections caused by cytolysin-producing strains compared with non-hemolytic strains [133]. The higher prevalence of *cylA* in human enterococci compared to environment-associated enterococci has also been observed by [128]. In addition, a surveillance study of surface contamination in small animal veterinary hospitals and another study on canine and feline enterococci both reported *cylA* as the least prevalent virulence gene, which is in line with our prevalence results in the Environmental and Animal sectors [30,32].

Phenotypically, β-hemolysis was not observed in all *cylA*-carrying isolates. Of the 13 isolates harboring *cylA*, only nine exhibited β-hemolytic activity (69.2%), indicating an incomplete genotype–phenotype correspondence between *cylA* detection and hemolytic phenotype already described by other authors [128,131]. Conversely, most β-hemolytic isolates carried the *cylA* gene (60%, 9/15), in line with a previously reported correlation between *cylA* genotype and hemolysis phenotype in animals and humans [16]. Together, these results highlight the importance of combining phenotypic with genotypic methods to assess hemolytic activity.

Overall, 33.8% (25/74) of total isolates had none of the tested virulence genes, representing 25.8% (8/31) of isolates in Humans, 36.4% (8/22) in Animals and 42.9% (9/21) in the Environment. Among isolates carrying at least one virulence gene (66.2%, 49/74), 57.1% (28/49) harbored multiple virulence genes, whereas only 4.1% of all isolates (3/74) carried the full virulence gene panel tested. These findings demonstrate that virulence-associated traits are not restricted to clinical enterococci, but are distributed across human, animal, and environmental sectors. From a One Health perspective, this overlap suggests the presence of similar enterococcal strains and pathogenicity determinants across interconnected settings, reinforcing the importance of integrated surveillance.

### 2.5. Integrative Analysis

Interpretation of these findings should consider several methodological limitations. The number of isolates within individual subsectors was limited, which may accentuate differences in resistance and MDR prevalence, virulence gene prevalence, and species distribution. Sampling was cross-sectional and geographically restricted, preventing inference on temporal trends or broader regional patterns. The inclusion of animals presenting with general clinical conditions, rather than enterococcal infections specifically, due to sampling constraints and sample availability, limits direct comparisons between the animal and human clinical cohorts. In addition, disk diffusion interpreted using CLSI breakpoints may limit direct comparison with studies using EUCAST criteria or broth microdilution, while culture-based isolation may favor robust species such as *E. faecalis* and underrepresent less tolerant enterococci [26,50,56,57]. Finally, it is important to highlight that our study design does not allow inference about directionality or transmission of resistance or virulence pathways between sectors.

Despite these constraints, the study provides an integrated assessment of species distribution, phenotypic AMR, and resistance gene prevalence across Human, Animal, and Environmental sectors. The predominance of *E. faecalis* across sectors is consistent with its role as a successful generalist and gastrointestinal commensal [12,39,40]. In contrast, the concentration of *E. faecium* and MDR phenotypes among Sick isolates supports its recognized association with healthcare-associated resistance [50,54,134].

Statistical analyses showed a clear association between health status (healthy/sick) and resistance in the Human sector (Table 2). The Cochran-Mantel-Haenszel (CMH) test showed that resistance was significantly higher in Sick Human isolates than in Healthy Human isolates (CMH OR = 5.354, 95% CI [2.939, 9.754]; p ≤ 0.001). On the other hand, no significant differences were observed between Healthy and Sick Animals according to the CMH test (*p* = 0.961) or among Environmental subsectors according to a binomial Generalized Linear Mixed-Model (GLMM) with a Likelihood-Ratio Test (LRT) (GLMM-LRT; *p* = 0.357). The absence of significant differences among the Animal and Environmental sub-sectors suggests that antimicrobial resistance is broadly distributed within these sectors, rather than being associated with specific subsectors. This pattern suggests a wide distribution of resistant enterococci between Healthy and Sick Animals, as well as among Public Transport Surfaces, Canteen Food, and Surface Water. In contrast, significant differences observed between Healthy and Sick Humans point to distinct selective pressures within the human sector. The higher resistance levels detected in the Sick Humans subsector likely reflect the greater exposure to antimicrobial agents in clinical settings, particularly considering that all antimicrobials evaluated in this study are approved for use in human medicine.

The pooled Fisher’s exact test, although widely reported in the literature, was used only as an exploratory analysis method because resistance outcomes across antibiotics were non-independent. CMH and GLMM-LRT were considered the primary inferential methods, as they better reflected the structure of the data [135,136] while the small sample size further supported a cautious and rigorous analytical approach. Although pooled Fisher testing suggested significant differences in resistance across One Health sectors, this result was not corroborated by the GLMM analysis (GLMM-LRT, *p* = 0.357), which accounted for non-independence across antibiotics. This might indicate that AMR was not confined to a single sector but was instead broadly distributed across the One Health continuum. This finding indicates that, while differences in resistance patterns can be observed between Healthy and Sick Humans, no significant overall sector effect was detected. This is very important from a One Health standpoint, as it can suggest that antibiotic resistance is widely distributed across One Health sectors.

Species-level analysis identified *E. faecium* as the main driver of resistance. Resistance was approximately five times more likely in *E. faecium* than in *E. faecalis* (CMH OR = 5.852, 95% CI [2.922, 11.719]; *p* = ${2.38\;\times \;10}^{-8}$), consistent with evidence identifying *E. faecium* as the enterococcal species most strongly associated with AMR across multiple drug classes [38,42,74]. The detection of resistance and MDR isolates in Public Transport Surfaces, Canteen Food, and Surface Water further supports the permeability of sector boundaries and reinforces the need for integrated One Health surveillance across clinical, animal, food-chain, and environmental reservoirs [5,17,26,27,34,67,68,76,137].

Utilizing the same statistical methodology, the same categories were tested for virulence genes (Table 3). In contrast with phenotypical resistance, no association between health status (healthy/sick) and presence of virulence genes can be seen in the Human sector (*p* = 0.249). No association was observed between any of the subsectors within their corresponding sector. However, between sectors, when accounting for the differences between subsectors using a binomial GLMM-LRT, the differences can be considered significant (*p* = 0.028). Looking further into the difference between sectors, we found a significant difference between the Human and Animal sectors (*p* = 0.035, GLMM-LRT OR = 0.4702, 95% CI [0.2524, 0.8760]) and the Human and Environmental sectors (*p* = 0.011, GLMM-LRT OR = 0.3803, 95% CI [0.1977, 0.7315]). The difference between Animal and Environmental sectors was not significant (*p* = 0.569, GLMM-LRT OR = 0.8089, 95% CI [0.3898, 1.6788]). This might reflect differences between adaptations to Human hosts and non-Human hosts, though this is not apparent in antibiotic resistance differences within the same sectors.

Focusing on the co-occurrence of multidrug resistance and virulence determinants, 34 of the 49 MDR isolates (69.4%) carried at least one virulence gene, whereas 15 (30.6%) did not harbor any of the virulence genes tested. The three isolates that possessed the full set of virulence determinants assessed were also MDR, representing 6.1% (3/49) of all MDR isolates. These findings indicate that virulence genes were common among MDR enterococci, suggesting that resistance and virulence traits may frequently coexist. However, the small number of isolates harboring all virulence genes suggests that the acquisition of multidrug resistance may not necessarily be accompanied by the accumulation of a broader virulence profile, indicating that these determinants may be subject to different selective pressures.

## 3. Materials and Methods

### 3.1. Sampling and Microbial Isolation

Enterococcal isolates were recovered from 66 samples obtained from Humans (n = 31 fecal samples from 14 Healthy Humans; 17 individuals with an enterococcal infection), Animals (n = 22 fecal samples from 10 Healthy and 12 out-patient Sick Animals in a veterinary teaching hospital) and Environment (n = 13 from 9 raw and cooked Canteen Food, 1 Surface Water and 3 Public Transport Surfaces). Samples were collected concurrently in all sectors between late 2022 and early 2024 in the Lisbon metropolitan area, Portugal.

Human fecal samples were self-collected by healthy human participants, while animal fecal samples (healthy cats and dogs, and sick outpatient dogs) were collected either by the pet tutors or by veterinary healthcare professionals. Samples from sick animals included only outpatient dogs, which had general clinical conditions (e.g., varied tumors, bone and tissue lesions), and healthy cats and dogs had no known clinical conditions. In cases of healthy animals housed together, fecal samples were collected independently and processed as a composite single sample representative of the household. Samples from humans with varied community-acquired and healthcare-associated enterococcal infections were processed by three partner healthcare entities (two hospitals and one clinical laboratory), and pure enterococcal isolates were provided in cryotubes. Surface water samples were collected and processed by a partner laboratory, and pure enterococcal isolates were provided in selective media plates. Samples from public transport environments were collected from multiple high-contact surfaces frequently touched by passengers, both within vehicles and at stations. Environmental samples were subsequently pooled into composite samples according to surface type and sampling location (e.g., handles, ATMs and ticket machines, similar foodstuffs, or surface water). General patient or individual data was collected (age, gender, geographic location, underlying condition/infection, antibiotic usage in the previous months) in samples from humans and animals whenever compatible with the policies of collaborator institutions (available in Supplementary Table S1). These variables were not considered during participant selection and therefore were not used as inclusion or exclusion criteria.

Fecal and food samples were diluted (1:10) in Maximum recovery broth (MRB) and enriched overnight at 37 ± 2 °C prior to isolation, whereas surface samples were enriched in MRB and spread plated and water was directly inoculated. Samples were inoculated in Slanetz-Bartley agar (SBA, Liofilchem, Roseto degli Abruzzi, Italy) and Slanetz-Bartley agar supplemented with vancomycin (SBAvan) at 6 μg/mL [16], and incubated aerobically at 37 ± 2 °C for up to 48 h. Approximately 20% of characteristic colonies were randomly selected and streaked onto Bile-Esculin Azide (BEA) agar (Liofilchem, Roseto degli Abruzzi, Italy) aiming for presumptive genus confirmation associated with the capacity to hydrolyze esculin [138]. Except when justified, a single presumptive enterococcal isolate per sample was selected and subcultured on Brain–Heart Infusion agar (BHI, Liofilchem, Roseto degli Abruzzi, Italy). Cultures were maintained on BHI at 4 °C for routine handling and preserved at −80 °C in BHI supplemented with 20% glycerol.

### 3.2. Microbial Diversity by RAPD-PCR

Genomic DNA extraction was performed from pure colonies using the boiling lysis method [139]. The supernatant obtained was used as template DNA for PCR.

Genetic diversity was assessed by RAPD-PCR using primers OPC15 (5′-GACGGATCAG-3′) and (GTG)_5_ (5′-GTGGTGGTGGTGGTG-3′) in independent reactions [16,140]. Amplification was performed on a thermocycler GTC96S (Cleaver Scientific, Rugby, UK), under the following conditions: 95 °C for 5 min; 35 cycles of 95 °C for 45 s, 57 °C for 45 s, and 72 °C for 45 s; followed by a final extension at 72 °C for 10 min. Replicates (10%) were randomly selected for reproducibility assessment purposes.

Amplification products were resolved on 1% agarose gels prepared in 0.5× Tris-Borate-EDTA buffer (4.5 × 10^−2^ M Tris, 4.5 × 10^−2^ M boric acid, and 1 × 10^−3^ M Na2EDTA, NYZTech, Lisboa, Portugal). Each well was loaded with 8 μL of amplification mixture, 2 μL of GelRed 10× (Biotium, Fremont, CA, USA), and 2 μL of bromophenol blue 6× (Merck KGaA, Darmstadt, Germany). Electrophoresis was performed at 90 V for 3 h, and results were observed using the ChemiDoc XRS+ device (Bio-Rad, Hercules, CA, USA) and Image Lab 6.1. software (Bio-Rad, Hercules, CA, USA) [16].

RAPD-PCR profiles were analyzed using BioNumerics version 6.6 (BioMérieux, Marcy-l’Étoile, France) as a composite dataset. After gel images normalization, similarities were calculated using the Pearson correlation coefficient, and dendrograms were constructed using the pair group method with the arithmetic mean (UPGMA) method with 3% tolerance. The resulting similarity matrices were averaged to construct a final dendrogram using the UPGMA method. Reproducibility estimates derived from replicate analyses (10 pairs) were used to guide cluster definition and the selection of representative isolates for downstream analyses. Sector-specific dendrograms were subsequently generated to support the selection of representative isolates while preserving balanced representation across One Health sectors and subsectors. Isolate selection followed a hierarchical decision process, prioritizing, in sequence, distinct RAPD-PCR electrophoretic profiles, the sector and subsector of origin, and the initial isolation medium (SBA or SBAvan). Isolates that did not cluster with others above the defined similarity threshold were treated as single-member clusters and retained as representatives for downstream analyses.

### 3.3. Molecular Species and Genus Identification

Species-level identification was performed by PCR, targeting species-specific regions of the *sodA* gene using primer pairs specific for *E. faecalis*, *E. faecium*, and *E. durans*, as well as for the genus *Enterococcus* [141,142,143]. Primer sequences and expected amplicon size are presented in Table 4. The reaction mixtures for each set contained 10 µL of NZYTaq II 2x Green Master Mix (NZYTech, Lisboa, Portugal) including NZYTaq II DNA polymerase, reaction buffer, deoxyribonucleotides (dNTPs), magnesium chloride (MgCl2), and additives, 7 µL of Milli-Q water (Millipore, Merck KGaA, Darmstadt, Germany), 2 µL of DNA and 0.5 µL of forward and reverse primer at 50 pmol/µL (STABVIDA, Caparica, Portugal). Considering all components, the reaction mixtures totaled 20 µL [16].

PCR products were resolved by 1.2% agarose gel electrophoresis (90 V for 2 h) as previously described [16]. In all reactions, *E. faecalis* ATCC^®^ 29212, *E. faecium* E300 and *E. durans* DSMZ^®^ 20633 were used as positive controls and sterile water was added as a negative control. Ten percent of the replicates were randomly selected to guarantee reproducibility.

### 3.4. Antimicrobial Susceptibility Testing

#### 3.4.1. Phenotypic Analysis

Antimicrobial susceptibility testing was performed using the Kirby–Bauer disk diffusion method using twelve antibiotics representing distinct classes and cellular targets. Listed by alphabetical order of classes, the following antimicrobial list was chosen considering its relevance for enterococcal infections in human medicine [63]: high-level gentamicin (120 μg), high-level streptomycin (300 μg), ampicillin (10 μg), teicoplanin (30 μg), vancomycin (30 μg), erythromycin (15 μg), linezolid (30 μg), chloramphenicol (30 μg), quinupristin-dalfopristin (15 μg), doxycycline (30 μg), levofloxacin (5 μg) and rifampicin (5 μg) [144].

Bacterial suspensions were prepared from overnight-grown cultures and adjusted to a turbidity equivalent to 0.5 McFarland scale using Ringer solution (Oxoid, Hampshire, UK). The suspensions were spread onto agar plates, and antibiotic disks (Liofilchem, Roseto degli Abruzzi, Italy) were equidistantly placed on the agar surface. Plates were incubated at 37 ± 2 °C for 24 h, and inhibition zones were interpreted according to CLSI breakpoints for *Enterococcus* spp. [144]. To assess reproducibility, 10% of isolates were analyzed in duplicate. *E. faecalis* ATCC 29212 was used as quality control.

Vancomycin resistance was further confirmed by agar dilution at 6, 16, and 32 μg/mL. The 6 μg/mL concentration was used as a screening concentration for vancomycin resistance, while 16 and 32 μg/mL represented the CLSI intermediate and resistant MIC thresholds, respectively, for *Enterococcus* spp. Plates were incubated at 37 ± 2 °C for 48 h, and results were interpreted according to CLSI criteria [144]. Only isolates with confirmed vancomycin resistance by both AST and agar dilution methods were subjected to MIC determination by Etest (Liofilchem, Roseto degli Abruzzi, Italy).

#### 3.4.2. Genotypical Analysis

The antimicrobial resistance reported in antimicrobial susceptibility testing, to gentamicin (*aacA-aphD*), ampicillin (*pbp5*), vancomycin (*vanA*, *vanB*, *vanC*), erythromycin (*erm(B)*), quinupristin-dalfopristin (*vat(D)*) and doxycycline (*tet(M)*) was confirmed by multiplex PCR [145,146,147,148,149,150]. ARGs were selected considering the resistance phenotypes identified by AST, prioritizing those most frequently observed in enterococci, and the clinical relevance of the corresponding antibiotics in human and veterinary medicine.

PCR preparation was equal to that described in Section 3.3. Primer sequences and respective amplicon size are presented in Table 5. All reactions included positive and negative controls. As positive controls, the following strains were used: *E. faecalis* V583 (*aacA-aphD*, *pbp5*, *vanB*)*; E. faecalis* H161 (*vanA*), *E. faecalis* H32 (*tet(M)*) and *Enterococcus* AR3-1V (*vanC*) from the culture collection of the Microbugs Research Team (Faculty of Veterinary Medicine, University of Lisbon). Additionally, 10% of isolates were analyzed in independent replicate reactions for quality control. Amplification conditions were as follows: 95 °C for 5 min, 35 cycles of 95 °C for 45 s, 55 °C for 45 s, 72 °C for 1 min; 72 °C for 5 min. PCR products were visualized on 1.2% agarose gels by electrophoresis (90 V for 2 h) as previously described [16].

### 3.5. Virulence Factors

#### 3.5.1. Hemolytic Activity

Metabolically active cultures were inoculated onto Columbia agar plates supplemented with 5% horse blood (Frilabo, Trofa, Portugal) and incubated anaerobically at 37 ± 2 °C for 48 h, using Oxoid Anaerogen^TM^ sachets (Thermo Fisher Scientific, Waltham, MA, USA). Hemolytic activity was evaluated after incubation based on the appearance of the agar around the colonies. A transparent halo was interpreted as β-hemolysis, a greenish halo as α-hemolysis (partial hemolysis), and the absence of any change in the medium as γ-hemolysis (non-hemolytic activity) [128]. *E. faecalis* MMH594 and *E. faecalis* ATCC 29212 were included as positive and negative controls, respectively. Reproducibility was guaranteed by 10% replicates randomly selected.

#### 3.5.2. Virulence Genes

Virulence gene screening was performed in all representative isolates by multiplex PCR, targeting genes associated with cell aggregation (*agg*), immune evasion (*esp*), gelatinase production (*gelE*), and cytolysin/hemolysin activity (*cylA*) [16].

PCR assays were performed using the same reaction mixture described in Section 3.3 and Section 3.4.2. Primer sequences and expected amplicon size are shown in Table 6. Positive (*E. faecalis* MMH 594) and negative (sterile water) controls were included in every run, and 10% of isolates were randomly selected for independent replicate testing. The amplification protocol consisted of an initial denaturation at 95 °C for 5 min, 35 cycles of 95 °C for 45 s, 55 °C for 45 s, and 72 °C for 45 s, and final extension at 72 °C for 5 min. PCR products were resolved on 1.2% agarose gels by electrophoresis (90 V for 2 h) and visualized as previously reported [16].

### 3.6. Statistical Analysis

Statistical analyses were performed in R version 4.5.1. Phenotypic resistance determined by AST was analyzed as a binary outcome, resistant/non-resistant, by antibiotic. Virulence gene occurrence was analyzed as a binary outcome, present/absent, by gene. For two-level comparisons, 2 × 2 Fisher’s exact tests were applied per antibiotic or per virulence gene. For comparisons involving more than two levels, Fisher–Freeman–Halton exact tests with Monte Carlo estimation, B = 100,000, were used. Multiplicity across antibiotics or virulence genes was controlled using Holm’s adjustment.

Overall associations across antibiotics or virulence genes were assessed using methods that account for repeated testing across traits. Two-level factors were analyzed using the CMH test stratified by antibiotic or virulence gene. Factors with more than two levels were analyzed using binomial GLMM tests, with significance assessed by LRT. In these models, antibiotic or virulence genes were included as an adjustment factor, as appropriate. Sector-level differences were analyzed using a logistic mixed-effects model with inference based on an LRT. Per-antibiotic and virulence gene sector effects were assessed using joint Wald tests derived from a binomial generalized linear mixed model. Pairwise sector comparisons were reported as model-adjusted odds ratios with 95% confidence intervals and Wald *p*-values. For comparability with previous studies, pooled Fisher’s exact tests, Pooled-Fisher, were also reported. All tests were two-sided with α = 0.05.

## 4. Conclusions

An integrated One Health approach is required to address the dissemination of AMR. This framework supports the surveillance of resistant strains and resistance determinants across human, animal, and environmental sectors, while also enabling evaluation of antimicrobial efficacy in clinical settings. In line with the aims and hypothesis of this study, *Enterococcus* spp. were recovered from all analyzed sectors, supporting the interconnected nature of these reservoirs and the potential circulation of AMR and virulence determinants across One Health compartments.

*E. faecalis* was the dominant species across all sectors, accounting for 85.1% of isolates and exceeding 66.7% in each environment. *E. faecium* was less prevalent but was mainly associated with Sick Human and Animal subsectors, consistent with its recognized role in clinical settings. Other *Enterococcus* spp. were primarily detected in environmental samples, reinforcing the ecological diversity of enterococci across interconnected reservoirs.

Resistance to clinically relevant antimicrobials was observed across all sectors, particularly for streptomycin, rifampicin, and quinupristin-dalfopristin. High resistance to the latter was reported in *E. faecium* and other *Enterococcus* spp., whereas rifampicin resistance likely reflects acquired determinants and selective pressures extending beyond human clinical use. The detection of VRE, especially among Sick Humans, remains particularly concerning given the importance of vancomycin as a last-resort antimicrobial.

Despite its lower prevalence, *E. faecium* exhibited higher resistance levels than *E. faecalis*, consistent with its enhanced capacity to acquire resistance determinants in healthcare-associated environments. This contributes to the emergence of MDR strains and increases the risk of dissemination across sectors, further supporting the hypothesis of ecological connectivity between human, animal, and environmental reservoirs.

Environmental sources reflected cumulative antimicrobial pressures from both human and animal origins and acted as important reservoirs of resistance. These settings remain underrepresented in current research, particularly high-contact public surfaces outside healthcare environments. Moreover, most studies continue to focus on isolated sectors, limiting the broader understanding of cross-sector transmission dynamics.

Overall, these findings support the study hypothesis and highlight the value of integrated One Health surveillance frameworks to characterize the distribution, persistence, and dissemination of AMR across interconnected reservoirs.

## Figures and Tables

**Table 1:** Distribution of resistance across One Health sectors and subsectors to each tested antibiotic.

Sector			Antibiotic											
	Subsector	CN n (%)	S n (%)	AMP n (%)	QDA * n (%)	C n (%)	TEC n (%)	VAN n (%)	VAN-MIC n (%)	E n (%)	LZD n (%)	LEV n (%)	RD n (%)	DXT n (%)
**Human**	Healthy (n = 14)/(n_QDA_ = 1)	0 (0.0)	2 (14.3)	0 (0.0)	1 (100.0)	1 (7.1)	0 (0.0)	2 (14.3)	0 (0.0)	3 (21.4)	0 (0.0)	0 (0.0)	8 (57.1)	0 (0.0)
	Sick (n = 17)/(n_QDA_ = 4)	4 (23.5)	5 (29.4)	11 (64.7)	2 (50.0)	0 (0.0)	2 (11.8)	10 (58.8)	9 (52.9)	14 (82.4)	0 (0.0)	10 (58.8)	11 (64.7)	1 (5.9)
**Animal**	Healthy (n = 10)/(n_QDA_ = 1)	0 (0.0)	2 (20.0)	0 (0.0)	1 (100.0)	1 (10.0)	0 (0.0)	2 (20.0)	0 (0.0)	6 (60.0)	0 (0.0)	2 (20.0)	5 (50.0)	0 (0.0)
	Sick (n = 12)/(n_QDA_ = 2)	2 (16.7)	2 (16.7)	0 (0.0)	1 (50.0)	0 (0.0)	1 (8.3)	3 (25.0)	0 (0.0)	5 (41.7)	0 (0.0)	2 (16.7)	8 (66.7)	0 (0.0)
**Environment**	Canteen Food (n = 9)/(n_QDA_ = 0)	0 (0.0)	5 (55.6)	0 (0.0)	0 (0.0)	0 (0.0)	0 (0.0)	1 (11.1)	0 (0.0)	0 (0.0)	0 (0.0)	0 (0.0)	3 (33.3)	0 (0.0)
	Surface Water (n = 6)/(n_QDA_ = 1)	0 (0.0)	4 (66.7)	0 (0.0)	0 (0.0)	0 (0.0)	0 (0.0)	1 (16.7)	0 (0.0)	0 (0.0)	0 (0.0)	0 (0.0)	3 (50.0)	1 (16.7)
	Public Transport Surfaces (n = 6)/(n_QDA_ = 2)	0 (0.0)	3 (50.0)	2 (33.3)	1 (50.0)	0 (0.0)	0 (0.0)	0 (0.0)	0 (0.0)	2 (33.3)	0 (0.0)	0 (0.0)	2 (33.3)	0 (0.0)
	**Total** (n = 74)	6 (8.1)	23 (31.1)	13 (17.6)	6/11 (54.5)	2 (2.7)	3 (4.1)	19 (25.7)	9 (12.1)	30 (40.5)	0 (0.0)	14 (18.9)	40 (54.1)	2 (2.7)

Antibiotics tested, by alphabetic order of classes: CN—Gentamicin, S—Streptomycin, AMP—Ampicillin, QDA—Quinupristin-dalfopristin, C—Chloramphenicol, TEC—Teicoplanin, VAN—Vancomycin, E—Erythromycin, LZD—Linezolid, LEV—Levofloxacin, RD—Rifampicin, DXT—Doxycycline. * Quinupristin-dalfopristin antimicrobial susceptibility results exclude *E. faecalis*, which is intrinsically resistant to this antibiotic. VAN column corresponds to disk-diffusion resistant results and VAN-MIC column refers to VRE whose resistance was confirmed by MIC determination (MIC ≥ 32 μg/mL).

**Table 2:** Summary of antibiotic resistance comparisons by category, considering sectors, subsectors and species.

Category	Sub-Category	Resistant Isolates (Total n = 192)	*p*-Value Pooled-Fisher	*p*-Value	Statistical Test	OR 95% Confidence Interval (CI)
Humans	Healthy	23	9.44 × 10^−8^	6.33 × 10^−9^	CMH	5.354 [2.939, 9.754]
	Sick	78				
Animals	Healthy	23	0.876	0.961	CMH	1.086 [0.535, 2.206]
	Sick	29				
Environment	Canteen Food	15	0.757	0.646	Binomial GLMM-LRT	-
	Surface Water	13				
	Public Transport Surfaces	11				
Sectors	Humans	101	0.016	0.357	Binomial GLMM-LRT	-
	Animals	52				
	Environment	39				
Species/genus	*E. faecalis*	149	9.17 × 10^−6^	1.83 × 10^−7^	Binomial GLMM-LRT	-
	*E. faecium*	29				
	*Enterococcus* spp.	14				
Accounted species	*E. faecalis*	149	1.43 × 10^−6^	2.38 × 10^−8^	CMH	5.852 [2.922, 11.719]
	*E. faecium*	29				
Sectors	Humans	101	0.031	0.426	Binomial GLMM-LRT	0.655 [0.231, 1.855]
	Animals	52				
	Humans	101	0.001	0.121	Binomial GLMM-LRT	0.460 [0.172, 1.227]
	Environment	39				
	Animals	52	0.249	0.487	Binomial GLMM-LRT	0.702 [0.258, 1.905]
	Environment	39				

Legend: Resistance total is the sum of resistant isolate–antibiotic outcomes within each category (overall n = 192). *p*-value (Pooled-Fisher) is the Fisher exact *p*-value computed after pooling counts across antibiotics. The adjacent *p*-value reports the overall comparison adjusting for antibiotic—using the Cochran-Mantel-Haenszel (CMH) for two-level contrasts and a binomial Generalized Linear Mixed-Model Likelihood-Ratio Test (GLMM-LRT) for factors with >2 levels. Significant value *p* < 0.050.

**Table 3:** Summary of virulence gene comparisons by category, considering sectors, subsectors and species.

Category	Sub-Category	Virulence Genes Identified (Total n = 87)	*p*-Value Pooled-Fisher	*p*-Value	Statistical Test	OR 95% Confidence Interval (CI)
Humans	Healthy	19	0.203	0.249	CMH	1.657 [0.788, 3.483]
	Sick	31				
Animals	Healthy	12	0.337	0.455	CMH	0.604 [0.224, 1.626]
	Sick	10				
Environment	Canteen Food	10	0.416	0.315	Binomial GLMM-LRT	-
	Surface Water	3				
	Public Transport Surfaces	5				
Sectors	Humans	50	0.006	0.028	Binomial GLMM-LRT	-
	Animals	22				
	Environment	18				
Species/genus	*E. faecalis*	75	0.642	0.563	Binomial GLMM-LRT	-
	*E. faecium*	4				
	*Enterococcus* spp.	8				
Accounted species	*E. faecalis*	75	0.449	0.498	CMH	0.582 [0.186, 1.823]
	*E. faecium*	4				
Sectors	Humans	50	0.027	0.035	Binomial GLMM-LRT	0.4702 [0.252, 0.876]
	Animals	22				
	Humans	50	0.004	0.011	Binomial GLMM-LRT	0.380 [0.198, 0.732]
	Environment	18				
	Animals	22	0.594	0.569	Binomial GLMM-LRT	0.809 [0.390, 1.679]
	Environment	18				

Legend: Virulence Genes total is the sum of the presence of Virulence genes-isolate outcomes within each category (overall n = 87). *p*-value (Pooled-Fisher) is the Fisher exact *p*-value computed after pooling counts across Virulence Genes. The adjacent *p*-value reports the overall comparison adjusting for Virulence Genes—using the Cochran-Mantel-Haenszel (CMH) for two-level contrasts and a binomial Generalized Linear Mixed-Model Likelihood-Ratio Test (GLMM-LRT) for factors with >2 levels. Significant value *p* < 0.050.

**Table 4:** Primers used for species and genus identification, including sequence and amplicon size.

Species/Genus	Primer	Primer Sequence (5′-3′)	Amplicon Size (bp)	Reference
*E. faecalis*	FL1 FL2	F-ACTTATGTGACTAACTTAACC R-TAATGGTGAATCTTGGTTTGG	360	[141]
*E. faecium*	FM1 FM2	F-GAAAAAACAATAGAAGAATTAT R-TGCTTTTTTGAATTCTTCTTTA	215	[141]
*E. durans*	DU1 DU2	F-GCATTATTACCAGTGTTAGTGGTTG R-TGAATCATATTGGTATGCAGTCCG	186	[142]
*Enterococcus* spp.	Ent1 Ent2	F-TACTGACAAACCATTCATGATG R-AACTTCGTCACCAACGCGAAC	112	[143]

**Table 5:** Primers used for the identification of ARGs, including sequence and product length.

Gene	Resistance to	Primer Sequence (5′-3′)	Amplicon Size (bp)	Reference
*aacA-aphD*	High-level gentamicin	F-GATTGCCAGAACATGAATTACACGA R-CATAACCACTACCGATTATTTCAAT	156	[150]
*erm(B)*	Erythromycin	F-GAAAAGGTACTCAACCAAATA R-AGTAACGGTACTTAAATTGTTTAC	639	[149]
*pbp5*	Ampicillin	F-CATGCGCAATTAATCGG R-CATAGCCTGTCGCAAAAC	444	[145]
*tet(M)*	Tetracycline, doxycycline	F-ACAGAAAGCTTATTATATAAC R-TGGCGTGTCTATGATGTTCAC	155	[147]
*vanA*	Vancomycin	F-TTGGGGGTTGCTCAGAGGAG R-CTTCGTTCAGTACAATGCGG	931	[150]
*vanB*		F-AAGCTATGCAAGAAGCCATG R-CCGACAATCAAATCATCCTC	536	[148]
*vanC*		F-GCAGGTTCTGCCTTATGTATGAA R-ATGAAATGGCGTCACAAGCA	339	[150]
*vat(D)*	Quinupristin-dalfopristin	F-GCTCAATAGGACCAGGTGTA R-TCCAGCTAACATGTATGGCG	271	[146]

**Table 6:** Virulence determinants tested, including gene, biological role in virulence, primers, amplicon size and reference.

Gene	Role in Virulence	Primer Sequence (5′-3′)	Amplicon Size (bp)	Reference
*agg*	Aggregation protein with a role in adherence to eukaryotic cells and cell aggregation and conjugation	F-CGGTACAGTTGGCAGTGTTTCG R-GGCTTGTGGGTCTTTGGCAGAG	775	[151]
*gelE*	Extracellular metalloendopeptidase. Hydrolyzes gelatin, collagen, hemoglobin and other compounds	F-ACCCCGTATCATTGGTTT R-ACGCATTGCTTTTCCATC	419	[29]
*cylA*	Activation of cytolysin which lyses a range of eukaryotic and Gram-positive cells	F-CGGGGATTGATAGGCTTCATCC R-TAACCATCTGGAAAGTCAGCAG	628	[151]
*esp*	Cell wall protein responsible for immune evasion. May be associated with the *cyl* genes located on a pathogenicity island	F-TTGCTAATGCTAGTCCACGACC R-GCGTCAACACTTGCATTGCCGAA	933	[29]

Adapted from Monteiro Marques et al. [17].

## Data Availability

The contributions presented in this study are included in the manuscript or as Supplementary Materials. Further inquiries can be directed to the corresponding author.

## Supplementary Materials

The following supporting information can be downloaded at: https://www.mdpi.com/article/10.3390/antibiotics15070657/s1, Supplementary Figure S1: Dendrogram constructed through RAPD-PCR using (GTG)5 and OPC15 primers, with the threshold average reproducibility shown as the red line; Supplementary Table S1: List of representative *Enterococcus* isolates, including One Health sector and subsector, and sampling of origin, general participant information, isolation media, enterococcal species, antimicrobial susceptibility testing and MIC (vancomycin) results, hemolytic activity, ARGs and virulence genes identified.

## Abbreviations

The following abbreviations are used in this manuscript:

AMR	Antimicrobial Resistance
ARGs	Antimicrobial Resistance Genes
AST	Antimicrobial Susceptibility Testing
BEA	Bile-Esculin Azide
BHI	Brain-Heart Infusion
CLSI	Clinical and Laboratory Standards Institute
CMH	Cochran-Mantel-Haenszel
EUCAST	European Committee on Antimicrobial Susceptibility Testing
GLMM	Generalized Linear Mixed-Model
GLMM-LRT	Generalized Linear Mixed-Model with Likelihood-Ratio Tests
HLGR	High-Level Gentamicin Resistance
LRT	Likelihood-Ratio Test
MDR	Multidrug-Resistant
MIC	Minimum Inhibitory Concentration
PCR	Polymerase Chain Reaction
RAPD-PCR	Random Amplification of Polymorphic DNA-Polymerase Chain Reaction
SBA	Slanetz-Bartley agar
SBAvan	Slanetz-Bartley agar supplemented with Vancomycin
UPGMA	Unweighted Pair Group Method with Arithmetic Mean
VRE	Vancomycin-Resistant Enterococcus
VREfm	Vancomycin-Resistant *Enterococcus faecium*
VVE	Vancomycin-Variable Enterococci
