# Supplementary material for: Cross-Sector Antimicrobial Resistance and Virulence in Enterococcus spp. from Humans, Animals and the Environment
Source: Antibiotics (Basel). 2026 Jul 2;15(7):657. doi: 10.3390/antibiotics15070657 (PMC13404461; doi:10.3390/antibiotics15070657)
Supplement: Supplementary file 1 [file antibiotics-15-00657-s001.zip › Supplementary Figure S1.pdf]

Supplementary Figure S1

Dendrogram constructed through RAPD-PCR using(GTG5)and(OPC15)primers, with the threshold average reproducibility shown as the red line

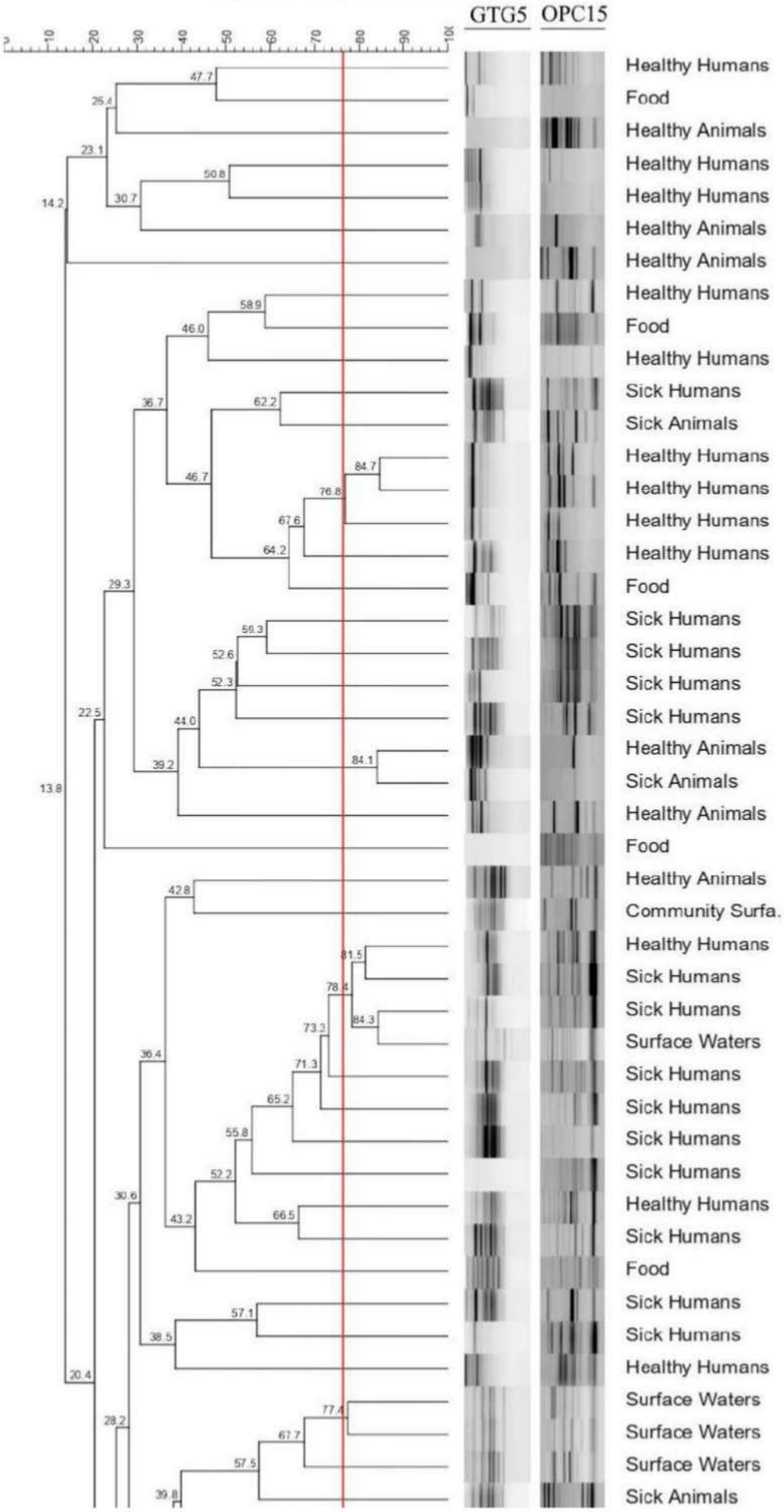

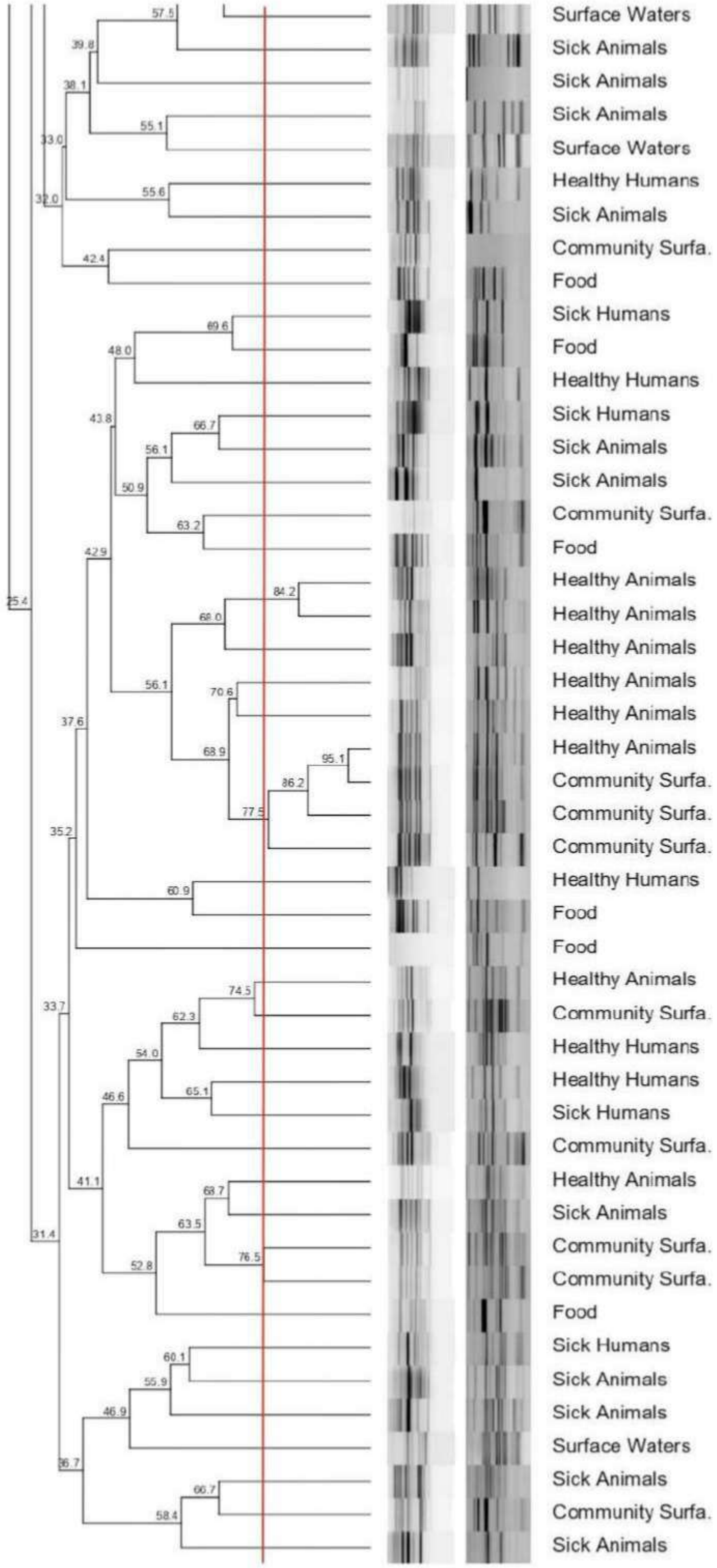

Surface Waters
Sick Animals
Sick Animals
Sick Animals
Surface Waters
Healthy Humans
Sick Animals
Community Surfa.
Food
Sick Humans
Food
Healthy Humans
Sick Humans
Sick Animals
Sick Animals
Community Surfa.
Food
Healthy Animals
Healthy Animals
Healthy Animals
Healthy Animals
Healthy Animals
Healthy Animals
Community Surfa.
Community Surfa.
Community Surfa.
Healthy Humans
Food
Food
Healthy Animals
Community Surfa.
Healthy Humans
Healthy Humans
Sick Humans
Community Surfa.
Healthy Animals
Sick Animals
Community Surfa.
Community Surfa.
Food
Sick Humans
Sick Animals
Sick Animals
Surface Waters
Sick Animals
Community Surfa.
Sick Animals
